# Zircon U–Pb chronology on plutonic rocks from northeastern Cambodia

**DOI:** 10.1016/j.heliyon.2021.e06752

**Published:** 2021-04-15

**Authors:** Naoto Kasahara, Sota Niki, Etsuo Uchida, Kosei Yarimizu, Rathborith Cheng, Takafumi Hirata

**Affiliations:** a Department of Resources and Environmental Engineering, School of Creative Science and Engineering, Waseda University, Ohkubo 3-4-1, Shinju-ku, Tokyo, 169-8555, Japan; b Geochemical Research Center, Graduate School of Science, The University of Tokyo, Hongo 7-3-1, Bunkyo-ku, Tokyo, 113-0064, Japan; c Department of Geology, Ministry of Mines and Energy, #79-80, St. 51, Sankat Phsar Thmey III, Khan Daun Penh, Phnom Penh, 12210, Cambodia

**Keywords:** U–Pb dating, Zircon, LA-ICP-MS, Plutonic rock, Cambodia

## Abstract

Zircon U–Pb geochronology was carried out on plutonic rocks from Phnom Daek, Phnom Koy Rmeas, Svay Chras, Kon Mom, Koh Nheak, Andong Meas, Oyadav South, Svay Leu, and Phnom Soporkaley. The zircon U–Pb ages from the plutonic rocks determined in this study can be roughly divided into two groups. One is the Late Permian to Triassic ages of 278–202 Ma for the Phnom Daek, Phnom Koy Rmeas, Oyadav South, Svay Leu, and Phnom Soporkaley, and the other is the early Cretaceous ages of 118–98 Ma for the Svay Chras, Kon Mom, Koh Nheak, and Andong Meas samples. The plutonic rocks from Phnom Daek, Phnom Koy Rmeas, Svay Leu, Oyadav South, and Phnom Soporkaley were likely formed by magmatic activity in the Loei Fold Belt. These plutonic rocks were likely formed in an extensional setting and/or a region where the continental crust was thin. The plutonic rocks of Svay Chras, Kon Mom, Koh Nheak, and Andong Meas were likely formed by magmatic activity in the Dalat-Kratie Fold Belt, related to the NW-directed subduction of the Paleo-Pacific Ocean plate. These plutonic rocks are thought to correspond to the Dinhquan suite in southern Vietnam. The Kon Mom and Koh Nheak plutonic rocks fall within the alkaline series, which suggests that the magma genesis was deep and far from the western Paleo-Pacific Ocean plate. Magmatic activity in the Dalat-Kratie Fold Belt migrated oceanward as a whole during the Cretaceous.

## 1 Introduction

**Figure 1:**
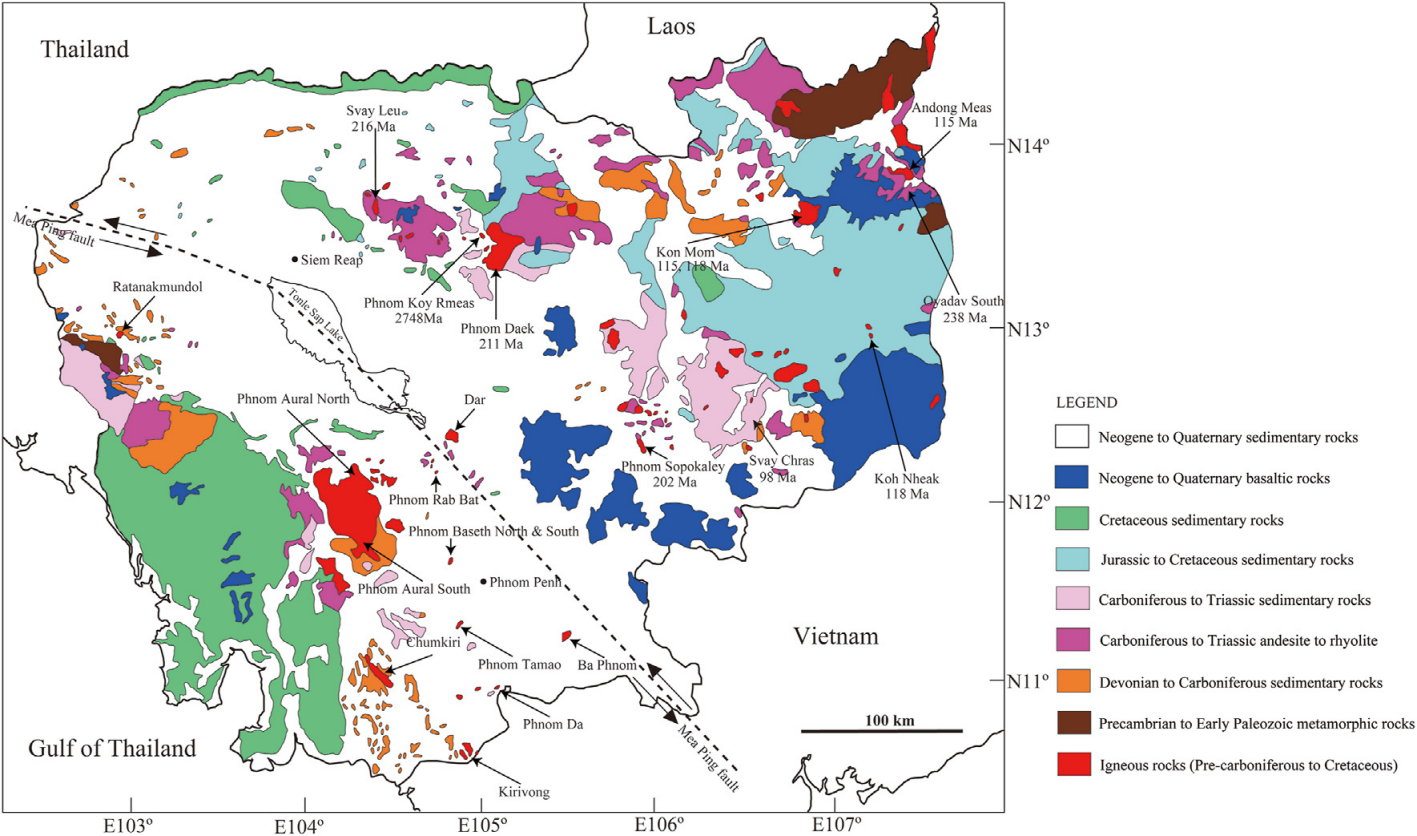
Simplified geologic map of Cambodia based on Cheng et al. (2019), showing the investigated plutonic rocks with zircon U–Pb ages.

The formation of Mainland Southeast Asia was induced by movement of the Indochina, South China, Sibumasu, West Myanmar, and Indian Continent terranes (Thuy et al., 2004b; Ferrari et al., 2008; Sanematsu et al., 2011; Metcalfe 2011, 2013; Searle et al., 2012; Morley, 2012; Morley et al., 2013; Shellnutt et al., 2013; Burrett et al., 2014; Kamvong et al., 2014; Zaw et al., 2014; Gardiner et al., 2015; Wang et al., 2016; Faure et al., 2018; Rossignol et al., 2018). Many plutonic rocks are distributed in Mainland Southeast Asia. These rocks formed as a result of collisions of the terranes and the subduction of the Paleo-Tethys, Meso-Tethys, and Ceno-Tethys Oceans, which existed between these terranes. Important metal deposits were also formed as a result of the movement of these terranes (Kamvong et al., 2014; Zaw et al., 2014; Gardiner et al., 2016; Nualkhao et al., 2018). Among the countries that make up Mainland Southeast Asia, research on plutonic rocks in Cambodia is lagging behind. Recently, Cheng et al. (2019) provided geochemical data on plutonic rocks throughout Cambodia and made tectonic considerations. According to Cheng et al. (2019), Cambodian plutonic rocks are divided into northeastern and southwestern regions by the Mae Ping Fault, which is inferred to extend from the northwest through Tonle Sap Lake to the south (Figure 1). Magnetic susceptibility, whole-rock chemical composition, and Sr-Nd isotope ratios were shown to be different for both regions. The evidence suggests that the plutonic rocks in the northeastern region are mantle-derived and belong to the magnetite series, whereas the plutonic rocks in the southwestern region have been greatly influenced by continental crust materials and belong to the ilmenite series. Rb–Sr isotope dating has been performed on rocks from both regions. The plutonic rocks in the southwestern region are rich in Rb and have low Sr contents, therefore, high-accuracy dating was successful with this technique. Conversely, because plutonic rocks in the northeastern region have low Rb contents and are rich in Sr, the accuracy of the Rb–Sr dating was low. In some cases, ages were obtained with data from multiple plutonic rocks along a straight line in the diagram, which is not a reliable technique.

U–Pb dating of zircon is a highly reliable method of dating igneous rocks (e.g., Dickin, 1995; Faure and Mensing, 2005; Schoene, 2014). Zircon does not contain primary Pb and is highly stable against weathering and metasomatism. The valences and ionic radii (eight coordinates) of Zr, U, and Pb are +4 and 0.84 Å, +4 and 1.00 Å, and +2 and 1.29 Å, respectively (Shannon and Prewitt, 1969). U can therefore substitute for Zr, whereas Pb is not initially incorporated into the crystal. As zircon is commonly present in rocks, these features of U and Pb allow the rocks to be accurately dated.

In this study, U–Pb age data were obtained from zircons collected from plutonic rocks of northeastern Cambodia using laser ablation inductively coupled plasma mass spectrometry (LA-ICP-MS). Based on the zircon U–Pb age data, we discuss the petrogenesis and tectonic setting of these Cambodian plutonic rocks.

A geological map of Cambodia is shown in Figure 1. Cambodia is surrounded by mountainous regions such as the Cardamom Highlands in the west, the Khorat Plateau in the north, and the Kontum Massif in the east. Central Cambodia is occupied by plains, which were formed by the Mekong River in the east and the Tonle Sap River in the northwest (Cheng et al., 2019). The inferred Mae Ping fault runs from Thailand in the northwest through Lake Tonle Sap in Cambodia to southern Vietnam (Morley et al., 2013). In northeastern Cambodia, Precambrian to Paleozoic metamorphic rocks are distributed in the region near the Vietnamese border. The Mesozoic sedimentary rocks are widely distributed except in the plains. In addition, Neogene to Quaternary basaltic rocks are widely distributed, especially in the southeast.

## 2 Methods

### 2.1 Sampling

Based on the geological map (Figure 1), we conducted sampling of plutonic rocks distributed in northeastern Cambodia, which is divided by the inferred Mae Ping fault. We collected fresh rock samples from nine plutonic bodies in Phnom Daek (CG202), Phnom Koy Rmeas (CG204), Svay Chras (CG206 and CG208), Kon Mom (CG209B and CG211), Koh Nheak (CG216), Andong Meas (CG218), Oyadav South (CG222), Svay Leu (CG413), and Phnom Soporkaley (CG415). Drilled core samples of the plutonic bodies in Koh Nheak, Andong Meas, and Oyadav South provided by Angkor Gold Corp. were used for zircon U–Pb dating. Magnetic susceptibility measurements (SM30, ZH Instruments Brno, Czech, Republic) were conducted for selected fresh rock samples.

### 2.2 Separation of zircon grains

Each rock sample was ground to a particle size of 250 μm or smaller using an iron mortar. The pulverized product was panned in water, and the residue was dried in an oven at 105 °C. After magnetic minerals were removed using a neodymium magnet, particles with a specific gravity of 2.85 or more were recovered by heavy liquid separation using a sodium polytungstate solution. Then, 20–40 zircon crystals were handpicked from the recovered material under a stereomicroscope and embedded in petropoxy resin that was thinly applied to a glass slide. The petropoxy resin was cured at 140 °C and subsequently polished first using water-resistant abrasive paper, # 1200 and # 2500, and then 3 μm and 1/4 μm diamond paste. The polishing process exposed the near-centers of the sample zircons.

### 2.3 Cathodoluminescence imaging

Cathodoluminescence (CL) images of the zircons were taken to assess the presence or absence of residual cores and growth textures. For this purpose, a CL detector (MonoCL3, Gatan, CA, USA) attached to a field emission scanning electron microscope (JSM-7001F, JEOL, Tokyo, Japan) installed at the Kagami Memorial Research Institute for Materials Science and Technology of Waseda University was employed.

### 2.4 Dating equipment

Instrumentation, operating conditions, and standard samples are summarized in Table 1. The U–Pb isotopic ratio measurements were conducted with the LA-ICP-MS installed at the Geochemical Research Center of the Graduate School of Science, The University of Tokyo. The multiple collector ICP-MS system used in this study was a Nu Plasma 2 (Wrexham, UK) equipped with six high-gain ion detectors. An in-house laser ablation system with a Yb: KGW femtosecond laser (Carbide, Light Conversion, Lithuania) was used. The pit size generated by the laser ablation system was approximately 10–15 μm. Laser ablation was performed in a helium gas atmosphere. After the addition of Ar make-up gas, the sample aerosols were transported into the ICP mass spectrometer. Three Daly collectors and three electron multipliers were employed as the detector (Hattori et al., 2017; Obayashi et al., 2017). The laser frequency was 2 Hz, and the integration time was 22 s.

**Table 1:** LA-ICP-MS instrumentation, operating conditions, and standard samples.

Samples	CG202, CG204, CG206, CG208, CG211 [1], CG218	CG209B, CG211 [2], CG216, CG222, CG413, CG415
**Laser ablation system**		
Model	In-house Laser Probe	
Laser type	Femtosecond Laser (CARBIDE, Light Conversion, Lithuania)	
Laser wavelength	257 nm	
Fluence	1–5 J cm^-3^	
Ablation pit size	10–15 μm	
Frequency	2 Hz	
Carrier gas	He and Ar make-up gas	
Pre-ablation	Single hole drilling (1 s)	50 × 50 μm square around sampling point
Sampling mode	Single hole drilling	

**ICP-MS instrument**		
Model	Nu plasma II HR-MC-ICP-MS (Nu Instruments, Wrexham, U.K.)	
Forward power	1300 W	
Ar make-up gas flow rate	0.87–0.90 L/min	
He gas flow rate	0.60–0.70 L/min	
Detection system	Mixed Faraday-multiple ion couting array	
Gas blanc integration time	22 s	
Integration time	22 s	
Monitor isotope	Three Daly collectors: ^206^Pb, ^207^Pb, ^238^U, ^235^U instead of ^238^U only for CG209B	
	Three electron multiplier: ^202^Hg, ^204^ (Hg + Pb), ^208^Pb	
	Faraday cup: ^232^Th	
^207^Pb/^206^Pb correction	^∗1^ NIST SRM 610 or NIST SRM 612	
Primary standard	^∗2^ Nancy 91500 zircon	
Secondary standard	^∗3^ GJ-1 zircon	

^∗1^Jochum and Brueckner (2008)^∗2^Wiedenbeck et al. (1995), and ^∗3^Jackson et al. (2004).

Prior to data acquisition, the laser was used to clean the surface and remove possible surface contamination of Pb. For samples CG209B, CG211 [2], CG216, CG222, CG413, and CG415, this pre-ablation was performed on a 50-μm square around each measurement point. During the measurements, ^202^Hg, ^204^(Hg + Pb), ^206^Pb, ^207^Pb, ^208^Pb, ^232^Th, and ^238^U were detected. ^204^Pb and ^235^U were calculated from the count of each detected mass number by the following method. ^204^Pb was calculated using Eq. (1), assuming that ^204^Pb was not detected in the gas blank measurement. ^235^U was calculated from Eq. (2), assuming that the natural abundance ratio of uranium is ^238^U/^235^U = 137.88 (Jaffey et al., 1971). For sample CG209B, ^235^U was detected directly using a Daly collector, and ^238^U was subsequently calculated using Eq. (2).

(1) P204b=(Hg+Pb)204−H202g×((Hg+Pb)204H202g)gasblank

(2) U235=U238×1137.88

NIST SRM 610 standard glass (^207^Pb/^206^Pb = 0.9096) or NIST SRM 612 standard glass (^207^Pb/^206^Pb = 0.9073) (Jochum and Brueckner, 2008) was used for gain calibration of the high-gain ion detectors. As a primary standard sample, Nancy 91500 standard zircon (^206^Pb/^238^U ratio: 0.17928 ± 0.00018, ^207^Pb/^206^Pb: 0.07556 ± 0.00032; Sakata et al., 2017) was used to correct the ^206^Pb/^238^U ratio. GJ-1 standard zircon (^206^Pb/^238^U age: 600.4 Ma; Jackson et al., 2004) was used as a secondary standard sample. The U–Pb isotopic analysis consists of three points on NIST SRM 610 standard glass or NIST SRM 612 standard glass, three points on Nancy 91500 standard zircon, one point on GJ-1 standard zircon, 13 points on zircon samples, three points on NIST SRM 610 standard glass or NIST SRM 612 standard glass, and three points on Nancy 91500 standard zircon.

After measurement, the sample was examined optically under both transmitted and reflected light to ensure that the measurement excluded resin, inclusions, and cracks. Finally, concordia diagrams (Wetherill, 1956) were created using IsoplotR (Vermeesch, 2018).

## 3 Brief petrological description of plutonic rocks and zircons

**Figure 2:**
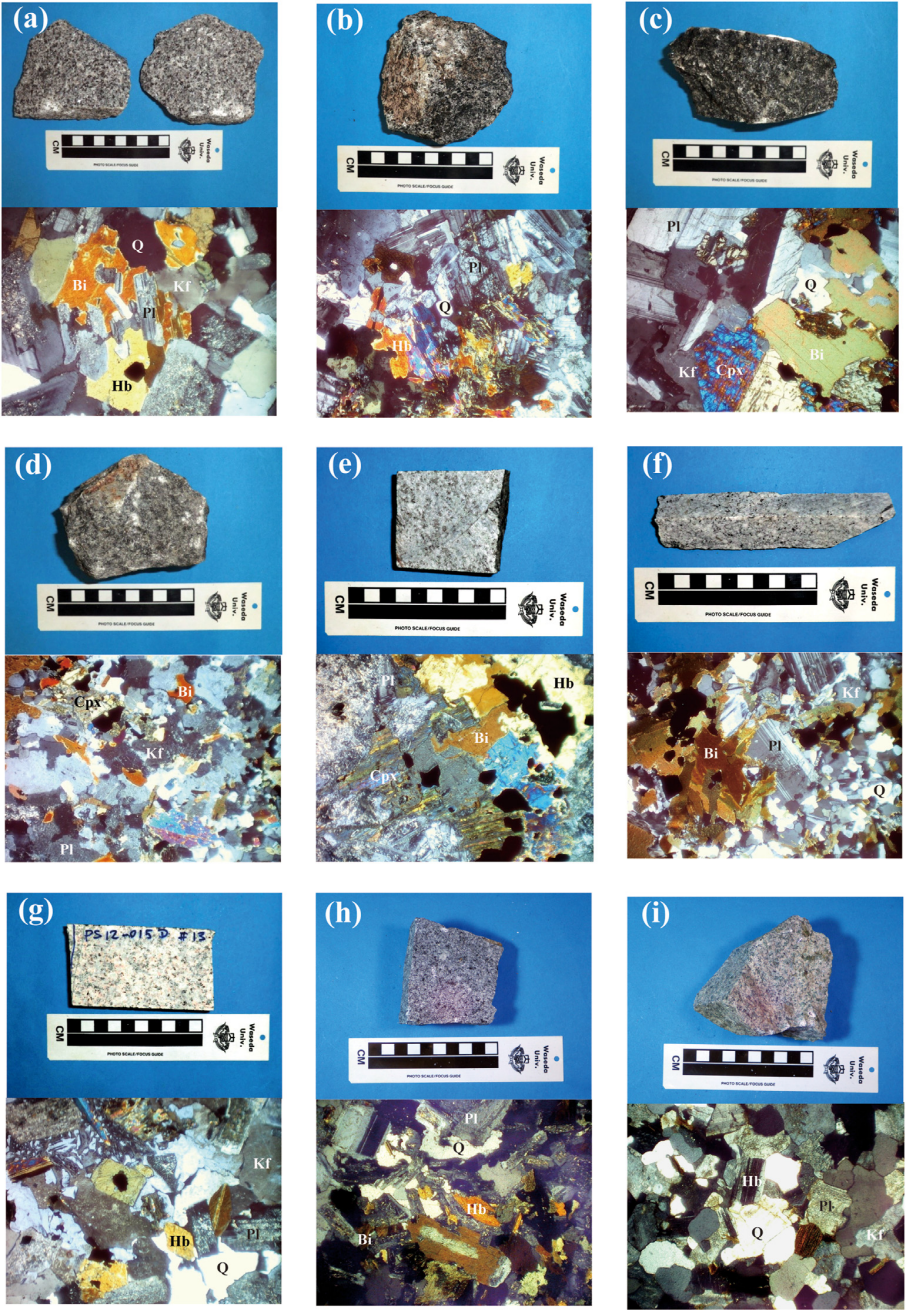
Photographs (above) and photomicrographs under crossed polars (below) of the representative plutonic rocks from NE Cambodia used for the zircon U–Pb dating. (a) granodiorite from Phnom Daek (CG202) (based on Cheng et al., 2019), (b) gabbro from Phnom Koy Rmeas (CG204), (c) syenite-diorite from Svay Chras (CG206), (d) syenite-diorite from Kon Mom (CG211), (e) gabbro from Koh Nheak (CG216), (f) granodiorite from Andong Meas (CG218), (g) granodiorite from Oyadav South (CG222), (h) granodiorite from Svay Leu (CG413), and (i) granodiorite from Phnom Soporkaley (CG415). *Abbreviations*: Pl, plagioclase, Q, quartz; Kf, potassium feldspar; Bi, biotite, Hb, hornblende; Cpx, clinopyroxene.

**Figure 3:**
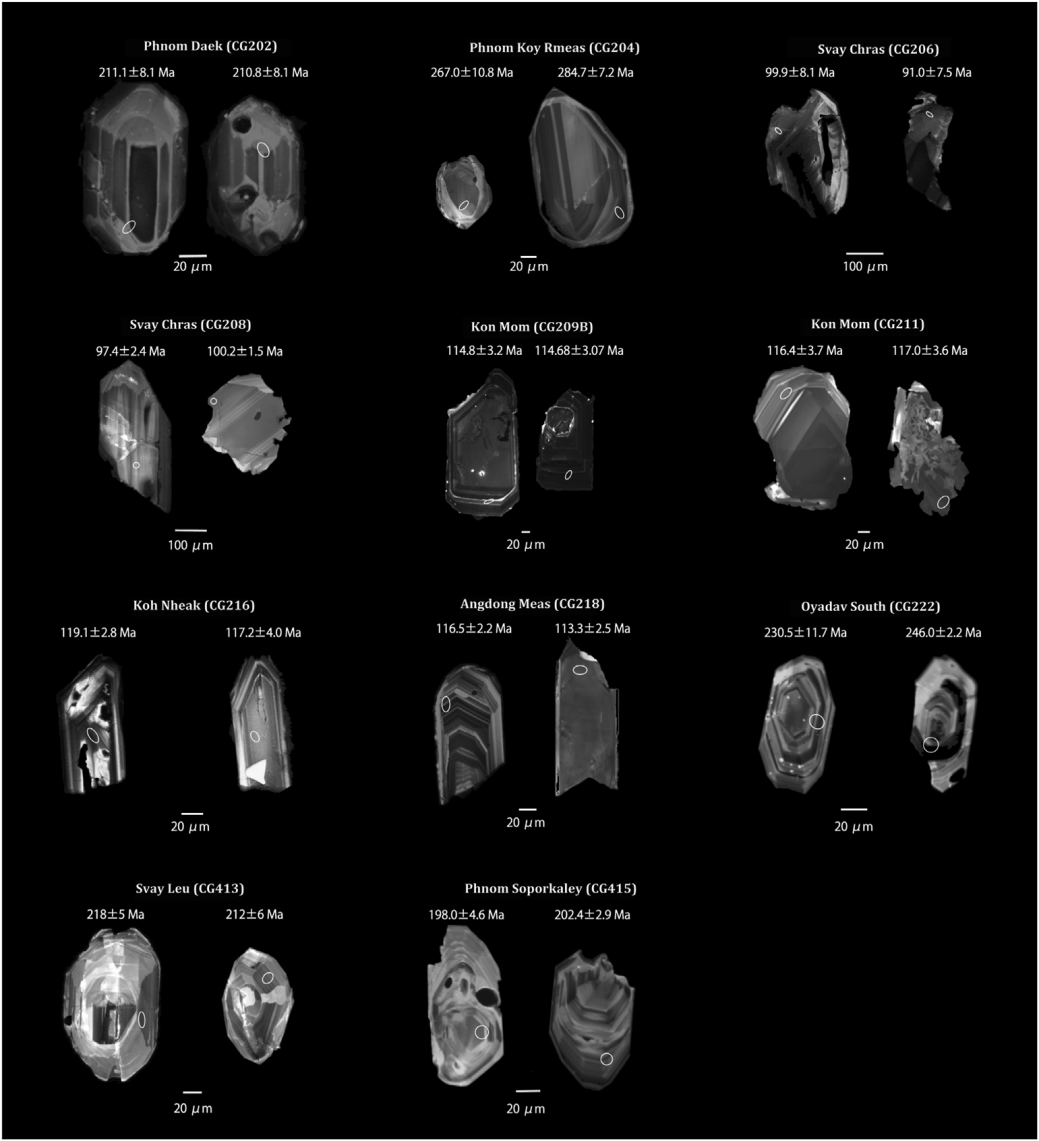
Representative CL images of zircon grains from plutonic rocks of northeastern Cambodia used for U–Pb dating. Circles show positions of analyzed points. The U–Pb ages of each zircon grain are (^206^Pb/^238^U) ages.

In this study, U–Pb dating was performed on zircon grains separated from 11 samples of nine plutonic rocks from northeastern Cambodia: Phnom Daek (CG202), Phnom Koy Rmeas (CG204), Svay Chras (CG206 and CG208), Kon Mom (CG209B and CG211), Koh Nheak (CG216), Andong Meas (CG218), Oyadav South (CG222), Svay Leu (CG413), and Phnom Soporkaley (CG415) (Figure 1). The samples of Koh Nheak (CG216), Andong Meas (CG218), and Oyadav South (CG222) were from cores provided by Angkor Gold Corp. Here we provide petrographic descriptions of each rock type and whole-rock thin section observations conducted with an optical microscope (Figure 2), as well as descriptions of zircons under a stereomicroscope and CL detector (Figure 3).

### 3.1 Description of rock samples and zircons

#### 3.1.1 Phnom Daek (granodiorite: CG202)

The main constituent minerals were plagioclase (30–40 vol.%), quartz (10–30 vol.%), hornblende (10–30 vol.%), biotite (10–30 vol.%), and potassium feldspar (2–10 vol.%). Titanite, apatite, zircon, and opaque minerals were identified as accessory minerals. Biotite was partly altered.

Zircon crystals often showed spindle shapes, and rare columnar crystals were observed. Particle size were 40–100 μm, and the color was light brown.

#### 3.1.2 Phnom Koy Rmeas (gabbro: CG204)

The main constituent minerals were plagioclase (30–40 vol.%), hornblende (30–40 vol.%), and quartz (2–10 vol.%). Opaque minerals were found as accessory minerals.

Zircon crystals often showed spindle shapes; rarely, columnar crystals were observed. They were 80–150 μm in size and showed a pale brown color.

#### 3.1.3 Svay Chras (syenite-diorite: CG206; syenite-diorite: CG208)

The main constituent minerals were plagioclase (30–40 vol.%), biotite (10–30 vol.%), pyroxene (10–30 vol.%), quartz (2–10 vol.%), potassium feldspar (2–10 vol.%), and amphibole (2–10 vol.%). Apatite and opaque minerals were confirmed as accessory minerals.

The zircon crystals in both samples CG206 and CG208 had irregular shapes, with particle sizes of 100–200 μm and clear to pale brown colors.

#### 3.1.4 Kon Mom (syenite: CG209B; syenite-diorite: CG211)

The main constituent minerals were potassium feldspar (30–40 vol.%), plagioclase (10–30 vol.%), biotite (10–20 vol.%), hornblende (10–20 vol.%), pyroxene (10–20 vol.%), and quartz (2–10 vol.%). Apatite and opaque minerals were confirmed as accessory minerals. Sample CG211 was relatively rich in zircons.

The zircon crystals in samples CG209B and CG211 had irregular shapes, with particle sizes of 150–250 μm and a dark brown color. Additionally, many cracks and inclusions were observed in the crystals, and metamictization occurred locally.

#### 3.1.5 Koh Nheak (gabbro: CG216)

The main constituent minerals were plagioclase (40–60 vol.%), hornblende (10–30 vol.%), and pyroxene (2–10 vol.%). Opaque minerals were identified as accessory minerals. Plagioclase was strongly altered.

The zircon crystals were columnar, with particle sizes of 50–200 μm, and a light brown color.

#### 3.1.6 Andong Meas (granodiorite: CG218)

The main constituent minerals were quartz (30–40 vol.%), plagioclase (30–40 vol.%), biotite (10–30 vol.%), and potassium feldspar (2–10 vol.%). Apatite and opaque minerals were confirmed as accessory minerals.

Zircon had columnar shapes, particle sizes of 40–300 μm, and a light brown color.

#### 3.1.7 Oyadav South (granodiorite: CG222)

The main constituent minerals were quartz (10–30 vol.%), plagioclase (10–30 vol.%), potassium feldspar (10–30 vol.%), and hornblende (10–30 vol.%). Titanite, epidote, and opaque minerals were identified as accessory minerals.

The zircon crystals were spindle-shaped, with particle sizes of 40–100 μm and dark brown color.

#### 3.1.8 Svay Leu (granodiorite: CG413)

The main constituent minerals were plagioclase (30–40 vol.%), quartz (10–30 vol.%), biotite (10–30 vol.%), and hornblende (10–30 vol.%). Titanite, epidote, and opaque minerals were identified as accessory minerals.

The zircon crystals were spindle-shaped or columnar, with particle sizes of 40–300 μm and a light brown color.

#### 3.1.9 Phnom Soporkaley (granodiorite: CG415)

The main constituent minerals were plagioclase (30–40 vol.%), quartz (10–30 vol.%), potassium feldspar (10–30 vol.%), hornblende (10–30 vol.%), clinopyroxene (2–10 vol.%), and biotite (2–10 vol.%). Epidote, tourmaline, and opaque minerals were identified as accessory minerals. Granophyric structures were frequently observed.

The zircon crystals had spindle shapes, with many defects in their peripheries. The particles were 50–150 μm in size and pale brown in color.

### 3.2 Geochemical background of the study area

The plutonic rocks show a wide range of SiO_2_ contents from 46 to 72 wt.%. They are classified as the magnetite series and show no negative Eu anomalies in the chondrite-normalized REE patterns, and biotite in the plutonic rocks has higher Mg/(Mg + Fe) molar fractions ranging from 0.45 to 0.7 (Cheng et al., 2019). The plutonic rocks from Koh Nheak, Svay Chras, and Kon Mom are classified as alkaline rocks, but the others are sub-alkaline rocks. The plutonic rocks from Svay Chras and Kon Mom are classified as A-type, but the others are I-type. Using Pearce's tectonic setting classification (Pearce et al., 1984), also according to Figure 10 of Cheng et al. (2019), the Kon Mom plutonic body falls within the syn-collision granite region, but the others fall within the volcanic arc granite region. The plutonic rocks from Phnom Daek, Svay Leu, Andong Meas, Koh Nheak, Oyadav South, Svay Chras, and Phnom Koy Rmeas show adakitic signatures.

## 4 Results

**Figure 4:**
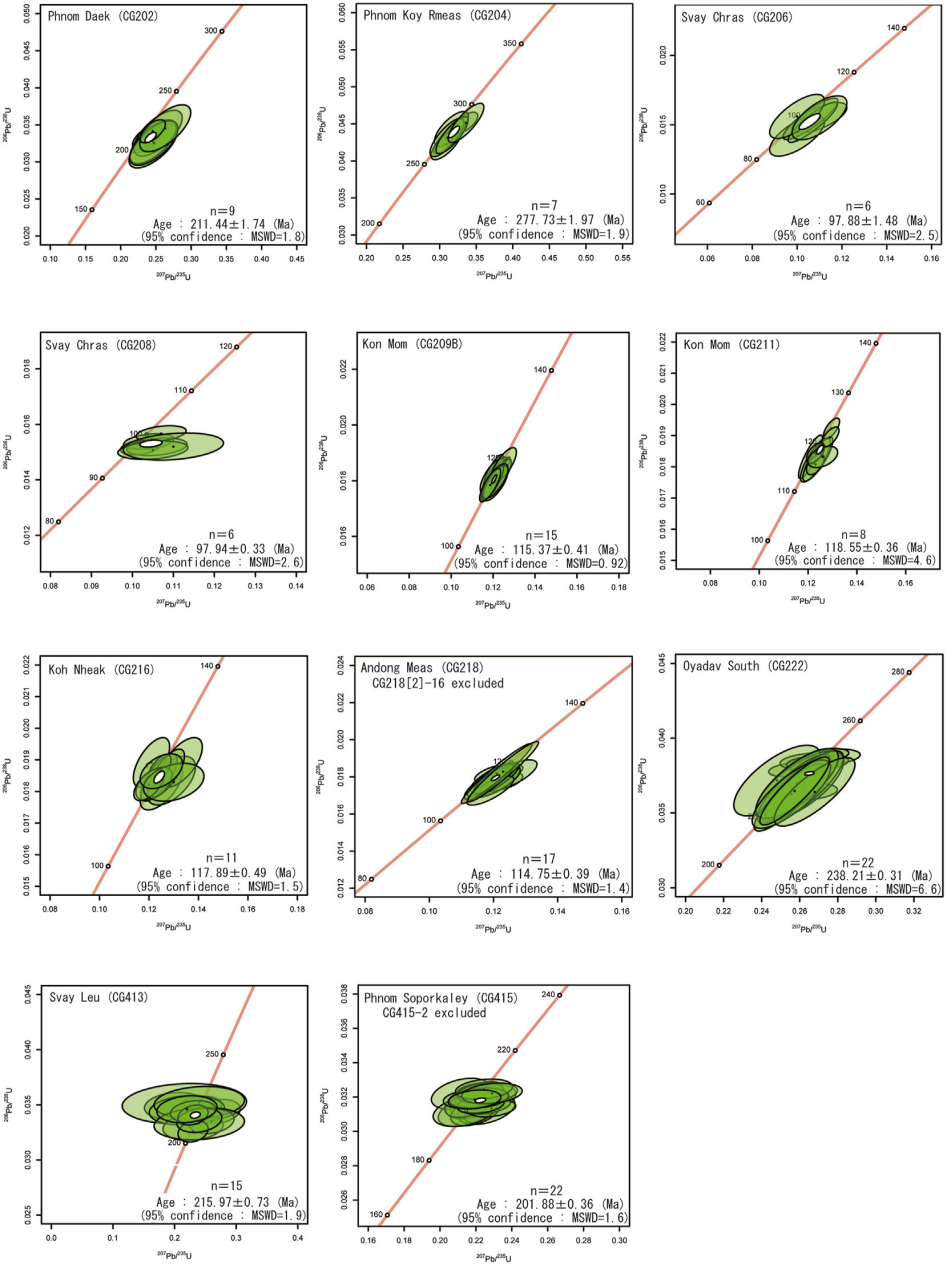
Concordia diagrams for zircon grains of plutonic rocks from northeastern Cambodia. *Abbreviation*: MSWD, mean square weighted deviation.

The measurement results are summarized in Appendix A, which details which zircon grains were included and excluded in the construction of the Wetherill concordia diagrams. For the Wetherill concordia diagrams constructed by IsoplotR (Vermeesch, 2018), the ^207^Pb/^235^U ratio is represented on the horizontal axis and the ^206^Pb/^238^U ratio is represented on the vertical axis; a zircon grain is defined as concordant if it has an error ellipse of 95% confidence (±2σ) that overlaps with the concordia curve. A zircon grain not on the concordia curve is a discordant sample (Figure 4). From the U–Pb isotopic data of concordant samples, the concordia ages were then calculated using the Wetherill concordia diagram. The concordia ages were determined from the two-dimensional weighted means of the ^207^Pb/^235^U ratio and the ^206^Pb/^238^U ratio according to the method described by Ludwig (1998).

*Phnom Daek (CG202)*: Nine concordant data were obtained, and the concordia age was determined to be 211.44 ± 1.74 Ma.

*Phnom Koy Rmeas (CG204)*: Seven concordant data were obtained. The concordia age was determined to be 277.73 ± 1.97 Ma.

*Svay Chras (CG206)*: Six concordant data were obtained, and the concordia age was determined to be 97.88 ± 1.48 Ma.

*Svay Chras (CG208):* Six concordant data were obtained, and the concordia age was determined to be 97.94 ± 0.33 Ma.

*Kon Mom (CG209B)*: Fifteen concordant data were obtained, and the concordia age was determined to be 115.37 ± 0.41 Ma.

*Kon Mom (CG211)*: Eight concordant data were obtained, and the concordia age was determined to be 118.55 ± 0.36 Ma.

*Koh Nheak (CG216)*: Eleven concordant data were obtained, and the concordia age was determined to be 117.89 ± 0.49 Ma.

*Andong Meas (CG218)*: Seventeen concordant data were obtained. Among the concordant samples, clear zoning, which is characteristic of other zircons in the sample of Andong Meas (CG218), could not be confirmed in the CL images of zircon sample CG218 [2]-16, which had a markedly different age value. This results suggests that this zircon was incorporated into the rock. Excluding these measurement data, the age was calculated using 16 concordant data. The obtained concordia age was 114.75 ± 0.39 Ma.

*Oyadav South (CG222)*: Twenty-two concordant data were obtained, and the concordia age was determined to be 238.21 ± 0.31 Ma.

*Svay Leu (CG413)*: Fifteen concordant data were obtained, and the concordia age was determined to be 215.97 ± 0.73 Ma.

*Phnom Soporkaley (CG415)*: Twenty-three concordant data were obtained, one (CG415-2) of which plotted away from the other data. From the 22 concordant data excluding this one datum, the concordia age was determined to be 201.88 ± 0.36 Ma.

## 5 Discussion

### 5.1 Comparison with whole-rock Rb–Sr isochron ages

Cheng et al. (2019) reported whole-rock Rb–Sr isochron ages for the same plutonic rocks presented here. The ages we obtained with U–Pb dating of zircons for Andong Meas (CG218), Oyadav South (CG222), Koh Nheak (CG216), Svay Chras (CG206 and CG208), and Phnom Soporkaley (CG415) are substantially different from the reported Rb–Sr ages. Andong Meas, Oyadav South, Koh Nheak, and Svay Chras are all located near the Vietnamese border. They plotted almost along the same line for the (^87^Sr/^86^Sr) vs. (^87^Rb/^86^Sr) diagrams, and an age of 332 Ma was obtained for all four locations from this isochron. However, U–Pb dating of zircons yielded ages of 115, 238, 118, and 98 Ma for Andong Meas (CG218), Oyadav South (CG222), Koh Nheak (CG216), and Svay Chras (CG206 and CG208), respectively, which are quite different from the age obtained from whole-rock Rb–Sr isochron dating.

Whole-rock Rb–Sr isochron dating for Phnom Soporkaley yielded an age of 118 Ma, but U–Pb dating of zircons also gave a substantially different age of 202 Ma (CG415). For the whole-rock Rb–Sr isochron dating, the age was obtained from two plutonic rock samples (CG414 and CG415). The two plots in the (^87^Sr/^86^Sr) vs. (^87^Rb/^86^Sr) diagram were close together, which may have prevented accurate chronology.

For Phnom Daek and Phnom Koy Rmeas, the Rb–Sr isochron age of 274 Ma was determined using data for both plutonic rocks. However, U–Pb dating of zircons, yielded ages of 211 Ma and 278 Ma for Phnom Daek (CG202) and Phnom Koy Rmeas (CG204), respectively.

### 5.2 (^143^Nd/^144^Nd)_i_ and (^87^Sr/^86^Sr)_i_ values obtained using zircon U–Pb ages

**Figure 5:**
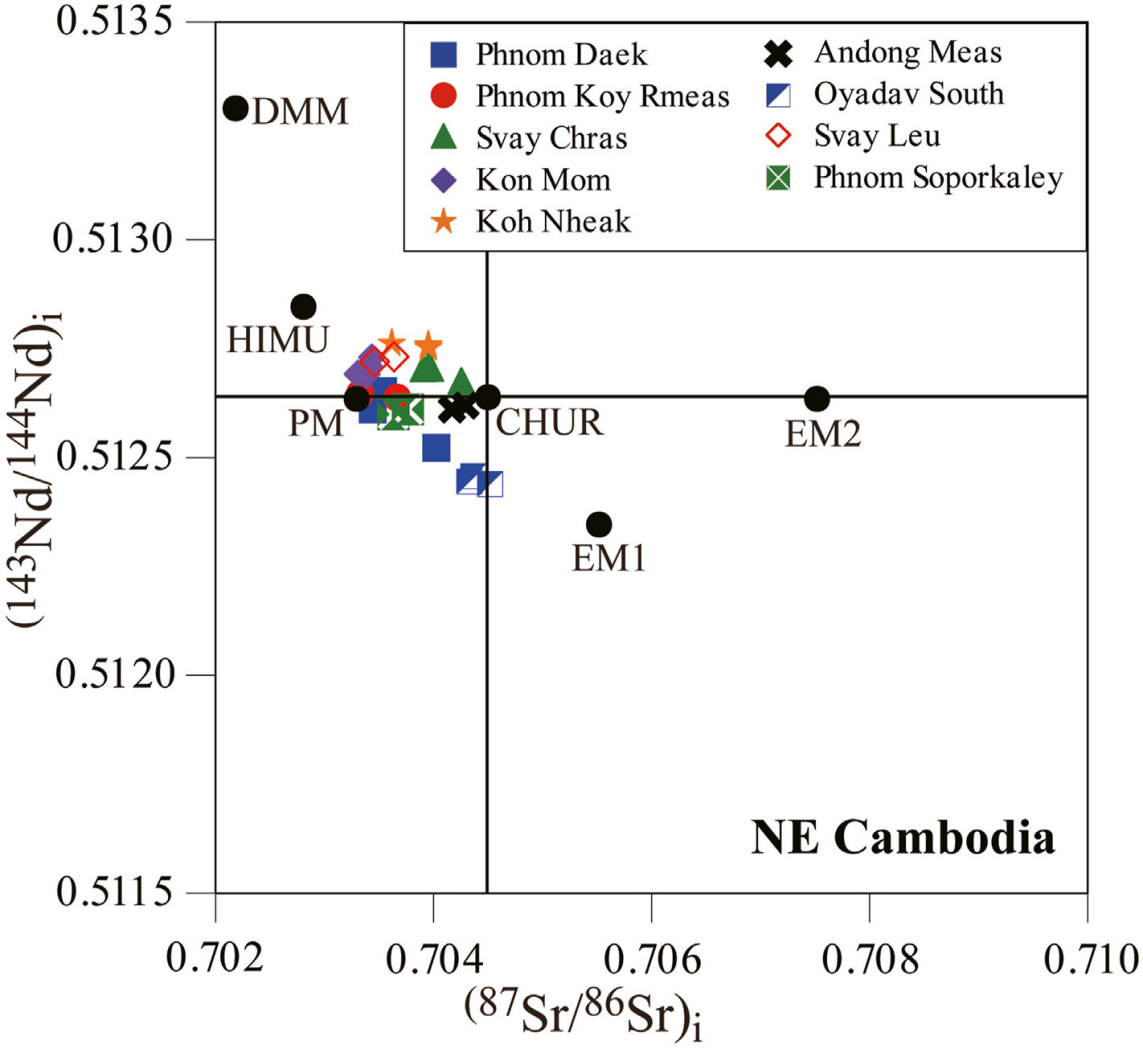
(^87^Sr/^86^Sr)_i_ vs. (^143^Nd/^144^Nd)_i_ diagram for plutonic rocks from northeastern Cambodia.

Cheng et al. (2019) measured ^143^Nd/^144^Nd and ^87^Sr/^86^Sr isotopic ratios. The (^143^Nd/^144^Nd)_i_ values and (^87^Sr/^86^Sr)_i_ values were recalculated from the present zircon U–Pb age data of the plutonic rocks in northeastern Cambodia. They ranged from 0.51244 to 0.51276 (εNd_i_ = +2.19 to +6.94) and from 0.7033 to 0.7045 (εSr_i_ = –14.89 to +1.66), respectively (Table 2). We then plotted (^143^Nd/^144^Nd)_i_ vs. (^87^Sr/^86^Sr)_i_ (Figure 5) for all plutonic rocks. They plotted near primitive mantle (PM) or chondritic uniform reservoir (CHUR) field (Schaefer, 2016). These results indicate that the plutonic rocks in northeastern Cambodia are of mantle origin, irrespective of age.

**Table 2:** Results of Sr and Nd isotope analyses for plutonic rocks from northeastern Cambodia (data obtained from Cheng et al., 2019) and their initial isotopic values calculated using zircon U–Pb ages obtained in this study.

Location	Sample No.	zircon U–Pb age	n	MSWD	^87^Sr/^86^Sr	±1σ	^87^Rb/^86^Sr	(^87^Sr/^86^Sr)i	^143^Nd/^144^Nd	±1σ	^147^Sm/^144^Nd	(^143^Nd/^144^Nd)i
Phnom Daek	CG102	211 .44 ± 1.74Ma	9	1.8	0.704200	0.000008	0.217685	0.703545	0.512861	0.000006	0.152183	0.512651
	CG201				0.705020	0.000006	0.327909	0.704034	0.512784	0.000004	0.190195	0.512521
	CG202				0.704741	0.000030	0.326095	0.703452	0.512856	0.000003	0.135938	0.512609
Phnom Koy Rmeas	CG204	277.73 ± 1.97 Ma	7	1.9	0.703727	0.000064	0.012592	0.703677	0.512957	0.000004	0.176536	0.512636
	CG205				0.703385	0.000008	0.012158	0.703337	0.513001	0.000005	0.195364	0.512646
Svay Chras	CG206	97.88 ± 1.48Ma	6	2.5	0.704443	0.000008	0.333461	0.703979	0.512787	0.000003	0.129547	0.512704
	CG207A				0.704288	0.000010	0.233853	0.703963	0.512791	0.000003	0.129602	0.512708
	CG207B				0.704385	0.000008	0.336699	0.703917	0.512789	0.000002	0.134479	0.512703
	CG208				0.704516	0.000008	0.180666	0.704264	0.512754	0.000003	0.122327	0.512675
Kon Mom	CG210	118.55 ± 0.36Ma	8	4.6	0.704926	0.000011	0.879173	0.703444	0.512825	0.000004	0.125272	0.512728
	CG211				0.705530	0.000007	1.316212	0.703312	0.512779	0.000003	0.114037	0.512690
	CG212				0.705023	0.000007	0.947877	0.703426	0.512811	0.000003	0.135402	0.512706
	CG213				0.705851	0.000007	1.502645	0.703319	0.512785	0.000004	0.120910	0.512691
	CG214				0.704896	0.000008	0.889414	0.703397	0.512790	0.000004	0.129992	0.512689
Koh Nheak	CG215	117.89 ± 0.49Ma	11	1.5	0.704118	0.000012	0.093692	0.703961	0.512897	0.000004	0.186621	0.512753
	CG216				0.704134	0.000008	0.103706	0.703960	0.512908	0.000004	0.193994	0.512759
	CG217				0.703744	0.000006	0.069993	0.703626	0.512899	0.000004	0.175806	0.512763
Andong Meas	CG218	114.75 ± 0.39Ma	17	1.4	0.704594	0.000006	0.179064	0.704302	0.512707	0.000003	0.118409	0.512618
	CG219				0.704540	0.000007	0.223377	0.704176	0.512691	0.000003	0.109363	0.512609
Oyadav South	CG220	238.21 ± 0.31Ma	22	6.6	0.706781	0.000009	0.665691	0.704526	0.512627	0.000006	0.121339	0.512438
	CG221				0.706693	0.000006	0.684722	0.704373	0.512644	0.000004	0.120576	0.512456
	CG222				0.706671	0.000007	0.688765	0.704337	0.512637	0.000003	0.124062	0.512444
Svay Leu	CG412	215.97 ± 0.73Ma	15	1.9	0.704669	0.000012	0.338882	0.703628	0.512899	0.000006	0.123197	0.512725
	CG413				0.703927	0.000013	0.145776	0.703479	0.512926	0.000004	0.144485	0.512722
Phnom Soporkaley	CG414	201.88 ± 0.36Ma	22	1.6	0.707216	0.000011	1.251637	0.703622	0.512792	0.000004	0.144201	0.512602
	CG415				0.706978	0.000014	1.110868	0.703789	0.512797	0.000004	0.146774	0.512603

### 5.3 Tectonic settings of the plutonic rocks in northeastern Cambodia

The U–Pb ages of zircons in 11 rock samples from nine plutonic rocks in northeastern Cambodia have been determined. The sampled plutonic rocks can be roughly divided into two age groups (Figure 1). One group is of Late Permian to Triassic age, 278–202 Ma, which includes the samples from Phnom Daek (CG202), Phnom Koy Rmeas (CG204), Oyadav South (CG222), Svay Leu (CG413), and Phnom Soporkaley (CG415). The other is of early Cretaceous age, 118–98 Ma, and includes the samples from Svay Chras (CG206 and CG208), Kon Mom (CG209B and CG211), Koh Nheak (CG216), and Andong Meas (CG218).

**Figure 6:**
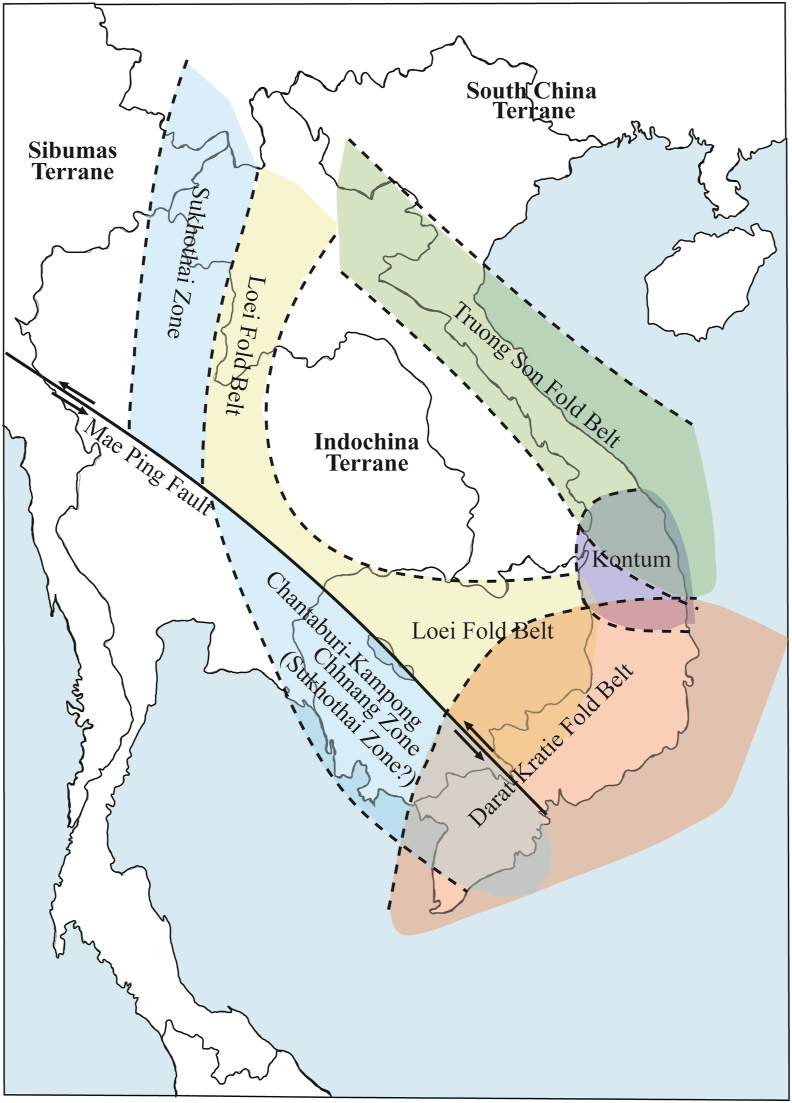
Simplified tectonic map of Mainland Southeast Asia. Data obtained from Metcalfe (2013), Khin Zaw et al. (2014), Cheng et al. (2019), and this study.

**Figure 7:**
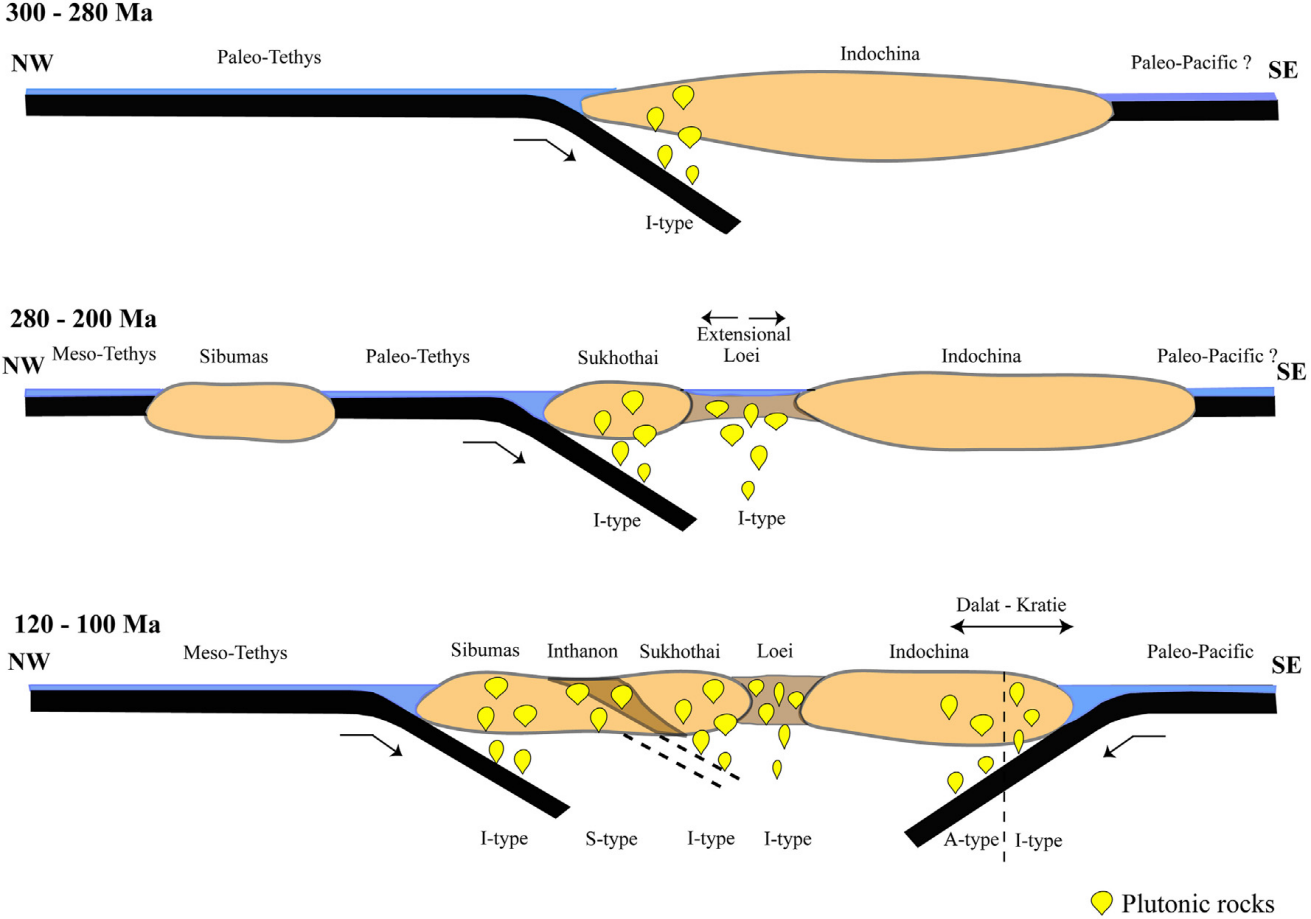
Schematic of the tectonic evolution of Mainland Southeast Asia during the periods of 300–280 Ma, 280–200 Ma, and 120–100 Ma.

The ages of the first group correspond to magmatic activity in the Loei Fold Belt (260–170 Ma) or Truong Son Fold Belt (290–190 Ma) (Zaw et al., 2009, 2014; Sanematsu et al., 2011; Kawakami et al., 2014; Manaka et al., 2014; Nualkhao et al., 2018). Based on their locations, the plutonic rocks of Phnom Daek, Phnom Koy Rmeas, Svay Leu, Oyadav South, and Phnom Soporkaley were likely formed by magmatic activity in the Loei Fold Belt, which likely extended north of the Mae Ping Fault in Thailand (Nualkhao et al., 2018) (Figure 6). This inference is supported by measurements of magnetic susceptibility and whole-rock chemistry (Cheng et al., 2019). Nd–Sr isotope ratios suggest that the Phnom Daek, Phnom Koy Rmeas, Oyadav South, Svay Leu, and Phnom Soporkaley samples are of mantle origin, and were less affected by continental crust materials (Cheng et al., 2019). These plutonic rocks were likely formed in an extensional tectonic setting and/or a region where the continental crust was thin. Therefore, it is reasonable to consider them to have been formed in the Loei Fold Belt, which was a back-arc basin formed during the eastward subduction of the Paleo-Tethys Ocean crust beneath the Indochina terrane (Figure 7).

In the Loei Fold Belt in Thailand, located north of the Mae Ping Fault, Au, Cu, Fe, and Sb deposits were formed in association with igneous rocks (Salam et al., 2014; Kamvong et al., 2014; Zaw et al., 2014; Nualkhao et al., 2018). Fe deposits are accompanied by plutonic rocks in Phnom Daek and Svay Leu. Exploration of Au and Cu deposits has been conducted in the Oyadav South plutonic rock. Mineralization in the Loei Fold Belt tends to be associated with adakitic rocks (Salam et al., 2014; Kamvong et al., 2014: Nualkhao et al., 2018; Cheng et al., 2019).

The early Cretaceous ages of the Svay Chras (CG206 and CG208), Kon Mom (CG209B and CG211), Koh Nheak (CG216), and Andong Meas (CG218) plutonic rocks suggest that they, along with Cretaceous granites in southwestern Cambodia, were formed as a result of the NW-directed subduction of the Paleo-Pacific Ocean plate in the east of southern Vietnam, beneath the Indochina terrane (Figure 7). These are classified as plutonic rocks belonging to the Dalat-Kratie Fold Belt (Thuy et al., 2004a, b) (Figure 6). Exploration of Au deposits has been conducted in the Koh Nheak and Andong Meas plutonic rocks. In the rock sample from Andong Meas (CG218), one zircon with an age of about 260 Ma was identified, but it was probably derived from plutonic xenoliths formed in the Loei Fold Belt.

Cretaceous granitic rocks in the Dalat Fold Belt in southern Vietnam were subdivided into the Dinhquan, Cana, and Deoca suites (Trung and Bao, 1980: Thang and Duyen, 1988). They are classified as calc-alkaline I-type granitic rocks (Thuy et al., 2004b). The Dinhquan suite consists mainly of hornblende-biotite granodiorite, diorite and minor granite. The Cana suite is composed of biotite granite poor in hornblende. The Deoca suite comprises pink porphyritic hornblende-biotite granodiorite, monzogranite, and diorite (Thuy et al., 2004a, b). The SiO_2_ content increases in the order of the Dinhquan suite < the Deoca suite < the Cana suite: 58–70 wt%, 68–77 wt%, and 73–78 wt%, respectively (Thuy et al., 2004b).

The Dinhquan suite shows no clear negative Eu anomaly in the chondrite-normalized REE pattern. Conversely, the Deoca suite shows a weak negative Eu anomaly, and the Cana suite shows a remarkable negative Eu anomaly (Thuy et al., 2004b). Additionally, the Dinhquan suite plots within the mantle array on the (^143^Nd/^144^Nd)_i_ vs. (^87^Sr/^86^Sr)_i_ diagram, but the Cana and Deoca suites plot outside of the mantle array (Thuy et al., 2004b), indicating contamination by continental crust materials.

Thuy et al. (2004a) estimated the emplacement ages of the Dinhquan, Deoca, and Cana suites using Rb–Sr mineral isochron and U–Pb zircon and titanite ages. The authors reported ages of the Dinhquan suite as ~112–100 Ma, the Cana suite as ~96–93 Ma, and the Deoca suite as ~92–88 Ma. From these data, the Svay Chras (CG206 and CG208), Kon Mom (CG209B and CG211), Koh Nheak (CG216), and Andong Meas (CG218) plutonic rocks (118–98 Ma) from the Dalt-Ktatie Fold Belt in northeastern Cambodia are thought to correspond to the Dinhquan suite in southern Vietnam. Thuy et al. (2004a) speculated that magmatic activity in the Dalat Fold Belt migrated oceanward as a whole. A similar oceanward migration of magmatic activity during the Yanshanian Orogeny from the Middle Jurassic to Cretaceous in southeastern China was reported by Jahn et al. (1976, 1990) and Guo et al. (1984). They suggested that this may have been related to the subduction angle increasing over time. The Andean-type magmatic activity of southeast China and southern Vietnam was separated by the collision of the Indian continent with the Eurasian plate (Tapponier et al., 1982, 1990; Hall, 2002, 2011).

The conclusion that the Svay Chras, Kon Mom, Koh Nheak, and Andong Meas plutonic rocks correspond to the Dinhquan suite is consistent with the above-mentioned conclusion of Thuy et al. (2004a). The Svay Chras, Kon Mom, Koh Nheak, and Andong Meas plutonic rocks have a wide range of SiO_2_ contents, from 46 to 68 wt%, show no negative Eu anomaly (Cheng et al., 2019), and plot within the mantle array on the (^143^Nd/^144^Nd)_i_ vs. (^87^Sr/^86^Sr)_i_ diagram (Figure 5). Unlike the Cretaceous granitic rocks in southern Vietnam, the Svay Chras, Kon Mom, and Koh Nheak plutonic rocks in northeastern Cambodia fall within the alkaline series (Cheng et al., 2019), which suggests that the magma genesis was deep, and far from the western Paleo-Pacific Ocean plate.

### 5.4 Improvements in the zircon U–Pb measurement by LA-ICP-MS

For samples of Phnom Daek (CG202), Phnom Koy Rmeas (CG204), Svay Chras (CG206 and CG208), Kon Mom (CG211 [1]), and Andong Meas (CG218), laser ablation points were determined using only CL images. For samples of Kon Mom (CG209B and CG211 [2]), Koh Nheak (CG216), Oyadav South (CG222), Svay Leu (CG413), and Phnom Soporkaley (CG415), laser ablation points were determined using both the CL and transmitted light images. Here we discuss improvements for determining appropriate measurement points based on these data.

Only information on the surfaces of the zircon grains can be obtained from the CL images; it is not possible to assess possible inclusions and cracks within the zircons that may affect the acquired data. When transmitted light images are used as well, appropriate laser ablation points can be found, based on both the surface and internal information of zircon grains. As shown in Appendix A, rock samples of Kon Mom (CG209B and CG211 [2]), Koh Nheak (CG216), Oyadav South (CG222), Svay Leu (CG413), and Phnom Soporkaley (CG415) had fewer excluded data than the others. This method is useful to determine the best possible measurement points and is important for future measurements.

The zircon grains from plutonic rock samples of Phnom Daek (CG202), Phnom Koy Rmeas (CG204), Svay Chras (CG206 and CG208), Kon Mom (CG211 [1]), and Andong Meas (CG218) were subjected to pre-ablation by a laser with a diameter of approximately 10–15 μm only at the measurement points. We also tested pre-ablating a 50 μm square around the measurement point for samples of Kon Mom (CG209B and CG211 [2]), Koh Nheak (CG216), Oyadav South (CG222), Svay Leu (CG413), and Phnom Soporkaley (CG415). Because the volatilization of the zircon surface by the laser is a result of heat, the area of volatilization is determined by heat propagation. For the zircon grains pre-ablated with the laser for 1 s at only the measurement point, some surface contamination is reflected in the acquired data. However, the data from zircon grains pre-ablated by a laser around the measurement point are not at all affected by surface contamination. This is because the surface contamination is removed from an area larger than the laser ablation radius. As shown in Appendix A, the plutonic rock samples treated this way, Kon Mom (CG209B and CG211 [2]), Koh Nheak (CG216), Oyadav South (CG222), Svay Leu (CG413), and Phnom Soporkaley (CG415), yielded fewer discordant data than the others. This suggests that the surfaces of the zircon grains were contaminated with Pb, and that it is preferable to perform pre-ablation over a wide area around the measurement point.

## 6 Conclusions

In this study, U–Pb dating of zircons using LA-ICP-MS was performed for nine plutonic rock samples from northeastern Cambodia. The results suggest the following:

(1) When determining the measurement points for U–Pb dating of zircon, it is necessary to acquire information on both the surfaces and interiors of the zircon grains, using transmitted light optical microscopy and CL images to avoid sampling cracked areas. Additionally, during pre-ablation, it is preferable to eliminate surface contamination from a wide area around the measurement points of zircon grains.
(2) The obtained U–Pb ages on the Wetherill concordia diagram of zircons for the plutonic rocks in northeastern Cambodia are: Phnom Daek: 211.44 ± 1.74 Ma (CG202), Phnom Koy Rmeas: 277.73 ± 1.97 Ma (CG204), Svay Chras: 97.99 ± 1.48 Ma (CG206) and 97.94 ± 0.33 Ma (CG208), Kon Mom: 115.37 ± 0.41 Ma (CG209B) and 118.55 ± 0.36 (CG211), Koh Nheak: 117.89 ± 0.49 Ma (CG216), Andong Meas: 114.75 ± 0.39 Ma (CG218), Oyadav South: 238.21 ± 0.31 Ma (CG222), Svay Leu: 215.97 ± 0.73 Ma (CG413), and Phnom Soporkaley: 201.88 ± 0.36 Ma (CG415). The sampled plutonic rocks can be roughly divided into two age groups. One group is of Late Permian to Triassic age, 278–202 Ma, and the other is of early Cretaceous age, 118–98 Ma.
(3) The plutonic rocks at Phnom Daek (CG202), Phnom Koy Rmeas (CG204), Svay Leu (CG413), Oyadav South (CG222) and Phnom Soporkaley (CG415) with ages of 278–202 Ma, were likely formed by magmatic activity in the Loei Fold Belt. These plutonic rocks probably occurred in an extensional tectonic setting and/or a region where the continental crust was thin. Fe, Cu, and Au mineralization accompanies the plutonic rocks in Phnom Daek, Svay Leu, and Oyadav South, which are adakitic in composition.
(4) The plutonic rocks at Svay Chras (CG206 and CG208), Kon Mom (CG209B and CG211), Koh Nheak (CG216), and Andong Meas (CG218), with ages of 118–98 Ma, were formed by magmatic activity in the Dalat-Kratie Fold Belt related to the NW-directed subduction of the Paleo-Pacific Ocean plate beneath the Indochina terrane. These plutonic rocks may correspond to the Dinhquan suite in southern Vietnam. The Svay Chras, Kon Mom, and Koh Nheak plutonic rocks fall within the alkaline series, which suggests that the magma genesis was deep, and far from the western Paleo-Pacific Ocean plate. Magmatic activity in the Dalat-Kratie Fold Belt migrated oceanward as a whole.

## Declarations

### Author contribution statement

Naoto Kasahara, Sota Niki, Kosei Yarimizu: Performed the experiments; Analyzed and interpreted the data; Contributed reagents, materials, analysis tools or data.

Sota Niki: Performed the experiments; Analyzed and interpreted the data; Contributed reagents, materials, analysis tools or data.

Etsuo Uchida: Conceived and designed the experiments; Wrote the paper.

Rathborith Cheng: Contributed reagents, materials, analysis tools or data.

Takafumi Hirata: Conceived and designed the experiments; Contributed reagents, materials, analysis tools or data.

### Funding statement

This research was supported financially in part by Grants-in-Aid for Scientific Research of the Japan Society for the Promotion of Science (E. Uchida: 16K06931, 19K05356; T. Hirata: A2624709). R. Cheng would like to express his sincere gratitude to the Japan International Cooperation Agency (JICA) for the master's scholarship opportunity and research grant.

### Data availability statement

Data included in article/supplementary material/referenced in article.

### Declaration of interests statement

The authors declare no conflict of interest.

### Additional information

No additional information is available for this paper.
